# Comparison of Gray Matter Atrophy in Behavioral Variant Frontal Temporal Dementia and Amyotrophic Lateral Sclerosis: A Coordinate-Based Meta-Analysis

**DOI:** 10.3389/fnagi.2020.00014

**Published:** 2020-02-11

**Authors:** Chunyan Luo, Na Hu, Yuan Xiao, Wenjing Zhang, Qiyong Gong, Su Lui

**Affiliations:** ^1^Functional and Molecular Imaging Key Laboratory of Sichuan Province, Department of Radiology, Huaxi MR Research Center (HMRRC), West China Hospital, Sichuan University, Chengdu, China; ^2^Psychoradiology Research Unit of the Chinese Academy of Medical Sciences (2018RU011), West China Hospital of Sichuan University, Chengdu, China

**Keywords:** amyotrophic lateral sclerosis, behavioral variant frontotemporal dementia, meta-analysis, gray matter, voxel-based morphometry

## Abstract

**Background:** There is growing evidence supporting behavioral variant frontotemporal dementia (bvFTD) and amyotrophic lateral sclerosis (ALS) as extreme points of a disease spectrum. The aim of this study was to delineate the common and different patterns of gray matter atrophy associated with bvFTD and with ALS by pooling together the results of previous voxel-based morphometry (VBM) studies.

**Methods:** We retrieved VBM studies that investigated gray matter atrophy in bvFTD patients vs. controls and in ALS patients vs. controls. Stereotactic data were extracted from those studies and subsequently tested for convergence and differences using activation likelihood estimation (ALE). A behavioral analysis using the BrainMap database was performed to assess the functional roles of the regions affected by bvFTD and/or ALS.

**Results:** Our study demonstrated a convergence of gray matter atrophy in the frontolimbic structures that involve the bilateral anterior insula and anterior cingulate cortex. Comparing the pattern of GM atrophy in bvFTD and ALS patients revealed greater atrophy in the frontomedial cortex, bilateral caudate, left anterior insula, and right thalamus in those with bvFTD and a higher degree of atrophy in the right motor cortex of those with ALS. Behavioral analysis revealed that the pattern of the affected regions contributed to the dysfunction of emotional and cognitive processing in bvFTD patients and the dysfunction of motor execution in ALS patients.

**Conclusion:** Our results revealed a shared neural basis between bvFTD and ALS subjects, as well as a specific and distinct neural signature that underpinned the clinical manifestations of those two diseases. Those findings outlined the role of the frontomedial-caudate circuit in the development of bvFTD-like deficits in ALS patients.

## Introduction

There is growing evidence supporting amyotrophic lateral sclerosis (ALS) and frontotemporal dementia (FTD) as extreme points of a disease spectrum. Although these two neurodegenerative diseases are characterized by extremely different spectra of symptoms, a partial clinical overlap is common (Lillo and Hodges, 2009; Cerami et al., 2015; Devenney et al., 2015). It has been estimated that 12–20% of patients with a diagnosis of FTD, especially those diagnosed as behavioral variant of FTD (bvFTD), can develop motor neuron dysfunction and are then diagnosed as ALS (Lomen-Hoerth et al., 2002; Burrell et al., 2011). On the other hand, behavioral changes and executive deficits, similar to those observed in bvFTD, have been well-described in ALS, which satisfies the diagnostic criteria for FTD (Strong et al., 2009; Lillo et al., 2010, 2012b; Phukan et al., 2012). Beyond the clinical features, pathological and genetic overlap exist between ALS and FTD: (i) trans-active response DNA binding protein of 43 kD (TDP-43) is the principal protein accumulated in ALS and in a subgroup of FTD cases (Neumann et al., 2006); (ii) an expanded GGGGCC hexanucleotide repeat in the chromosome 9 open reading frame 72 (C9orf72) gene has been identified in both FTD and ALS (DeJesus-Hernandez et al., 2011; Renton et al., 2011). Such a strong overlap indicates that neural correlates of both syndromes should also be related and highlights the need to elucidate whether and where there are common and different cerebral deficits in those with bvFTD and ALS.

Over the past decades, neuroimaging techniques have offered powerful measurement methods for assessing subtle changes in brain structure and function *in vivo*. Voxel-based morphometry (VBM) is a fully automated whole-brain technique allowing voxel-wise comparisons of morphological changes without confinement to specific regions, and it has been widely used in neurodegenerative disorders, including ALS and bvFTD. Gray matter volume reduction has been revealed in various brain areas in bvFTD, mainly including frontal and temporolimbic regions (Rosen et al., 2002; Boccardi et al., 2005; Seeley et al., 2008; Hornberger et al., 2011). On the other hand, VBM studies of ALS revealed gray matter volume abnormalities mainly in the motor cortices (Agosta et al., 2007; Mezzapesa et al., 2007; Thivard et al., 2007; Cosottini et al., 2012), though significant brain atrophy was not consistently found (Sage et al., 2007; Minnerop et al., 2009; Luo et al., 2012; De Marco et al., 2015). Reviewing the literature concerning VBM studies of bvFTD (Pan et al., 2012; Schroeter et al., 2014) and ALS (Sheng et al., 2015; Shen et al., 2016) revealed gray matter atrophy in overlapping frontal and temporolimbic regions, suggesting a neurostructural overlap between ALS and bvFTD. These findings can partially explain the overlap of signs and symptoms observed in clinical practice, as well as the co-occurrence of the two diseases. However, without a direct comparison, it remains unclear whether there is a true systematic overlap between the brain structural abnormalities in bvFTD and ALS and how these two diseases are different from each other.

To date, a very limited number of experimental studies have directly compared the patterns of gray matter atrophy in ALS and bvFTD, and there is some discrepancy between the results obtained (Lillo et al., 2012a; Crespi et al., 2018). A more detailed quantification of the convergent and different patterns of gray matter atrophy between bvFTD and ALS is yet to be reported. Indeed, there is an abundance of independent literature that has successfully delineated the patterns of gray matter atrophy associated with each disease by comparing each disease population to matched healthy controls. With additional primary VBM studies of ALS and bvFTD published in recent years, it is worthwhile to conduct an updated neuroimaging meta-analysis to look into the commonality and differences in brain structures impacted by ALS and bvFTD.

Therefore, we conducted a coordinate-based analysis of likelihood estimate (ALE) meta-analysis (Laird et al., 2009) in the current study, comparing VBM studies of bvFTD to those of ALS. Due to the very limited number of studies that have directly compared ALS and bvFTD, only studies that compared each patient population to healthy controls were considered for the present meta-analysis. In addition, a behavioral analysis based on the Brainmap database was performed to assess the functional roles of the atrophic regions.

## Methods

### Search Strategy

In accordance with the Preferred Reporting Items for Systematic Reviews and Meta-Analyses (PRISMA) statement (Moher et al., 2009), studies included in this meta-analysis were collected by a search of the PubMed, EMBASE, ISI Web of Science, and MEDLINE databases in June 2019. Keywords for the search were as follows: (i) ((“frontotemporal lobar degeneration”) OR (“behavioral variant frontotemporal dementia”) OR “FTD”) AND ((“voxel-based morphometry”) OR “VBM”); (ii) ((“amyotrophic lateral sclerosis”) OR “ALS”) AND ((“voxel-based morphometry”) OR “VBM”). The reference lists of retrieved articles and review articles were checked to obtain additional publications.

### Study Selection

A study was included if it: (1) was published in English with a peer review, (2) compared groups of ALS/bvFTD subjects with healthy control groups, (3) acquired images on an MR scanner with a minimum tesla strength of 1.5 and used the VBM procedure for high-resolution T1-weighted images to investigate alterations in whole-brain structure, and (4) used thresholds for significance that were either corrected for multiple comparisons or uncorrected with spatial extent thresholds. The following types of studies were excluded: (1) case reports, letters to the editor, meta-analysis, or review studies reporting no original data, (2) correlational studies, (3) studies that mainly investigated subjects with bvFTD/ALS plus other diagnoses, (4) studies that did not report the results of gray matter changes in Talairach or Montreal Neurological Institute (MNI) stereotactic space, (5) studies that included less than seven patients in each group, and (6) studies with a sample that overlapped with those of another publication. In cases with a sample overlap, the study with the largest sample size was selected. For studies that used multiple independent patient samples and separately compared them with the same healthy control sample, the results were regarded as separate datasets. If adequate information was not available in the original manuscript, the corresponding authors were contacted by e-mail to obtain additional details.

### Activation Likelihood Estimation (ALE) Meta-Analysis

Two investigators independently searched the literature, retrieved articles, and extracted data from each study. If an agreement was not obtained, then another author mediated. The opinion of the majority was adopted for the final analysis. The peak coordinates of the brain atrophy reported in the eligible studies constituted the meta-analysis input. The coordinates that were originally reported in the MNI spaces were converted into Talairach spaces within the GingerALE package using a transform called icbm2tal that was developed by Lancaster (Lancaster et al., 2007). The data from the Talairach coordinates were saved in a text file and entered into the GingerALE (Version 3.0.2; http://brainmap.org/ale/).

The current meta-analysis used the latest ALE algorithm (Eickhoff et al., 2012), which was implemented in GingerALE. To be specific, the ALE algorithm modeled the reported coordinates as the center peaks of the three-dimensional Gaussian probability distributions. A modeled activation (MA) map was then computed by combining the probability distributions of each focus and served to summarize the results of the study-specific localization probabilities. The spatial uncertainty associated with the activation foci was estimated with respect to the sample size from each study. The ALE image is a union of all of the MA maps. Such analyses formed a null distribution of the spatial extent on which to test whether the spatial coordinates reported across multiple studies represent stable and reliable gray matter atrophy.

A two-step analysis plan was conducted: first-level ALE analyses were separately performed for the two sets of data extracted from the bvFTD vs. healthy control studies and ALS vs. healthy control studies, with an initial threshold of voxel-level *p* < 0.005 and a minimum cluster size of 50 mm^3^. Next, the ALE-results of the above analysis were put into a second-level conjunction/contrast analysis testing our main concern: the patterns of commonality and difference between bvFTD and ALS. Second-level analyses involved quantitative conjunction analysis and non-parametric permutation simulations (10,000 permutations) to draw statistical inferences of differences between the ALS and bvFTD subjects. The statistical significance was corrected for multiple comparisons with the false discovery rate (FDR) at *q* < 0.05, with a cluster-extent threshold of 50 mm^3^ excluding spurious clusters of small size. Results were visualized with Mango (Version 4.0.1, http://www.nitrc.org/projects/mango), and the anatomical locations of the resulting coordinates were then determined using an anatomical atlas (Rorden and Brett, 2000).

### Analysis of Behavioral Domain Profiles

To assess the functional roles of those regions affected by ALS and/or bvFTD, behavioral analysis was performed using a plugin application for Mango. The behavioral decoding was based on the BrainMap database (http://www.brainmap.org/), in which functional imaging experiments were behaviorally classified according to five main categories (action, cognition, emotion, interception, and perception) and their related subcategories (Lancaster et al., 2012). Specifically, clusters from the above ALE meta-analysis were extracted as regions of interest (ROIs), and the probabilities for a brain ROI were determined for the 51 behavioral sub-domains. The probabilities are represented by the probability that reported behavior-specific activation foci fall within the ROI and increases as the ROI size increases. A size-adaptable expected probability was then calculated for significance testing. The null hypothesis that the observed probability of the activation foci was not different from the expected probability was tested using a binomial test. An effect-size z-score for each behavioral sub-domain was calculated (Lancaster et al., 2012). Only behavioral sub-domains with positive z-scores ≥ 3.1 were considered significant (*p* ≤ 0.05 with Bonferroni correction for multiple comparisons). This analysis tested which types of tasks were more likely than by chance to activate the atrophic regions identified in ALE analysis.

## Results

### Study Selection and Characteristics

**Figure 1:**
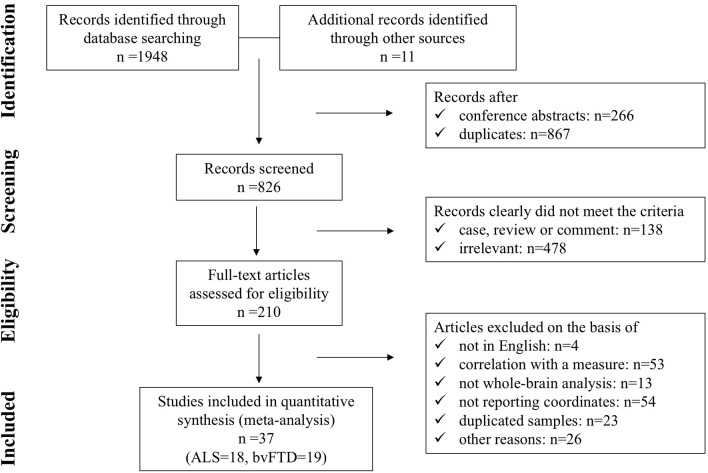
Flow diagram showing the identification and exclusion of studies in the meta-analysis.

A flow diagram showing the identification and exclusion of studies is provided in Figure 1. Ultimately, a total of 37 studies were included in the meta-analysis, including 19 on bvFTD (one study included three bvFTD groups) (Rosen et al., 2002; Grossman et al., 2004; Boccardi et al., 2005; Kanda et al., 2008; Seeley et al., 2008; Ash et al., 2009; Kipps et al., 2009; Libon et al., 2009; Pardini et al., 2009; Hornberger et al., 2011; Farb et al., 2013; Massimo et al., 2013; Lagarde et al., 2015; Yokoyama et al., 2015; Dermody et al., 2016; Mandelli et al., 2016; Melloni et al., 2016; Buhour et al., 2017a; Bertoux et al., 2018) and 18 on ALS (Chang et al., 2005; Grosskreutz et al., 2006; Agosta et al., 2007; Mezzapesa et al., 2007; Thivard et al., 2007; Grossman et al., 2008; Cosottini et al., 2012; Tedeschi et al., 2012; Meoded et al., 2013; Cerami et al., 2014; Zhang et al., 2014; Devine et al., 2015; Raaphorst et al., 2015; Tavazzi et al., 2015; Zhu et al., 2015; Buhour et al., 2017b; Kim et al., 2017; Christidi et al., 2018). Our final sample comprised 388 bvFTD patients (estimated mean age: 63.62, estimated mean disease duration: 54.97 months), 433 ALS patients (estimated mean age: 56.18, estimated mean disease duration: 27.26 months), and 1,016 HC (586 from bvFTD vs. HC studies, estimated mean age: 67.27; and 430 from ALS vs. HC studies, estimated mean age: 53.97). The demographic information and clinical characteristics of the included studies are shown in Table 1.

**Table 1:** Studies included in the meta-analysis.

**References**	**Comparison**	**Control**		**Patients**		**Disease duration (Mean ± SD)**	**ALSFRS-R score (Mean ± SD)**	**MMSE score (Mean ± SD)**	**MR scanner**	**Software**
		**Numbers**	**Age (Mean ± SD)**	**Numbers**	**Age (Mean ± SD)**					
Chang et al. (2005)	ALS vs. Con	22	44.5 ± 10.4	10	49.9 ± 8.0	28.8 ± 30	8.0 ± 7.1	29.8 ± 0.6	1.5	SPM2
Grosskreutz et al. (2006)	ALS vs. Con	17	58 ± 9.4	17	61 ± 13	24 ± 9	40 ± 6	NS	1.5	SPM
Agosta et al. (2007)	ALS vs. Con	18	52.2	25	54.1 [27–75]^*^	39 [6–58]^*^	29 ± 4.3	NS	1.5	SPM2
Mezzapesa et al. (2007)	ALS vs. Con	9	51.8 ± 12.7	16	58.6 ± 10.2	38.1 [8–89]^*^	27.4 ± 8.6	26.6 ± 2.9	1.5	SPM2
Thivard et al. (2007)	ALS vs. Con	25	44.9 ± 12.4	15	51.8 ± 8.7	30.9 ± 15.9	30 ± 6	NS	1.5	SPM2
Grossman et al. (2008)	ALS vs. Con	16	NS	26	59.0 ± 10.6	51.86 ± 47.0	37.0 ± 10.6	NS	3	SPM2
Cosottini et al. (2012)	ALS vs. Con	16	50.6 ± 10.9	20	58.0 ± 8.9	20.1 ± 17.5	38.2 ± 16.2	NS	1.5	FSL
Tedeschi et al. (2012)	ALS vs. Con	20	62.1 ± 8.5	20	60.7 ± 11.1	NS	34.2 ± 9	NS	3	SPM8
Meoded et al. (2013)	ALS vs. Con	17	59.2 ± 5.8	13	51.0 ± 8.3	20 ± 18	35.5 ± 4.3	NS	3	SPM8
Cerami et al. (2014)	ALS vs. Con	20	61.9 ± 7.9	14	59.1 ± 9.8	23.9 ± 20.7	36.8 ± 5.52	NS	3	SPM8
Zhang et al. (2014)	ALS vs. Con	43	51.8 ± 9.4	43	53.5 ± 9.0	16.9 ± 16.5	30.1 ± 6.6	28.2 ± 1.9	3	FSL
Devine et al. (2015)	ALS vs. Con	17	56 ± 13	ALS^a^:15	59 ± 13	23.8 ± 6.4	40 ± 4	NS	3	FSL
				ALS^b^:15	56 ± 11	28.7 ± 10.5	39 ± 6			
Raaphorst et al. (2015)	ALS vs. Con	21	60.7 ± 11.2	26	60.7 ± 12.5	23.3 ± 11.3	41.5 ± 3.7	NS	3	SPM5
Tavazzi et al. (2015)	ALS vs. Con	31	47.9 ± 14.5	20	54.5 ± 9.1	30.6 ± 18.3	34.5 [17–40]^*^	NS	1.5	FSL
Zhu et al. (2015)	ALS vs. Con	22	51.9 ± 6.9	22	47.86 ± 6.9	20.3 ± 14.0	39.6 ± 5.7	NS	3	SPM8
Buhour et al. (2017b)	ALS vs. Con	37	61.1 ± 11.1	37	61.2 ± 11.1	NS	37.3^#^	NS	3	SPM5
Kim et al. (2017)	ALS vs. Con	57	51.1 ± 5.2	62	52.7 ± 10.1	28.1 ± 19.0	37.3 ± 6.5	NS	3	SPM12
Christidi et al. (2018)	ALS vs. Con	22	59.5 ± 5.6	17	62.2 ± 10.7	14.2 ± 14.2	39.1 ± 6.3	NS	3	SPM8
Rosen et al. (2002)	bvFTD vs. Con	10	62.3^#^	10	61.8^#^	NS	NS	23.3 ± 4.4	1.5	SPM99
Grossman et al. (2004)	bvFTD vs. Con	12	68.5 ± 9.4	14	63.1 ± 12.2	42.4 ± 33.5	NS	18 ± 6.5	1.5	SPM99
Boccardi et al. (2005)	bvFTD vs. Con	26	69 ± 8	9	62 ± 5	30 ± 15	NS	14 ± 8	1.5	spm99
Kanda et al. (2008)	bvFTD vs. Con	20	65.2^#^	13	64.9^#^	NS	NS	17.7^#^	1.5	SPM2
Seeley et al. (2008)	bvFTD vs. Con	45	68.3 ± 7.9	bvFTD^c^: 15	65.9 ± 8.3	69.6 ± 57,6	NS	26.7 ± 0.7	1.5	SPM2
	bvFTD vs. Con			bvFTD^d^: 15	64.3 ± 8.9	56.4 ± 43.2	NS	23.3 ± 3.1		
	bvFTD vs. Con			bvFTD^e^: 15	62.3 ± 10.3	73.2 ± 38.4	NS	15.7 ± 7.9		
Ash et al. (2009)	bvFTD vs. Con	31	NS	12	64.8 ± 13.2	49.2 ± 19.2	NS	25.3 ± 5.5	1.5/3	SPM5
Kipps et al. (2009)	bvFTD vs. Con	12	66.4 ± 4.9	11	62.1 ± 6.6	62.4 ± 44.4	NS	25.1 ± 2.8	NS	SPM5
Libon et al. (2009)	bvFTD vs. Con	43	NS	51	61.3 ± 10.6	42.6 ± 31.1	NS	23.1 ± 6.8	3/1.5T	FSL
Pardini et al. (2009)	bvFTD vs. Con	14	NS	22	60.3 ± 8.3	56.4 ± 38.4	NS	NS	1.5	SPM5
Hornberger et al. (2011)	bvFTD vs. Con	18	64.8 ± 5.3	14	59.3 ± 7.9	44.4 ± 31.2	NS	24.9 ± 3.8	3	FSL
Farb et al. (2013)	bvFTD vs. Con		67.2 ± 1.2	8	66.7 ± 2.5	NS	NS	NS	3	SPM
Massimo et al. (2013)	bvFTD vs. Con	30	64.4 ± 10.3	37	63.7 ± 9.5	75.7 ± 40.3	NS	23.6 ± 0.9	NS	SPM5
Lagarde et al. (2015)	bvFTD vs. Con	18	67.8 ± 5.2	18	69.7 ± 9.7	64.8 ± 42	NS	25.6 ± 3.3 3	3	SPM8
Yokoyama et al. (2015)	bvFTD vs. Con	179	68.5	24	60.6 [29–83]^*^	NS	NS	NS	1.5/3/4	SPM8
Dermody et al. (2016)	bvFTD vs. Con	22	68.2 ± 6.7	24	63 ± 8.7	41.5 ± 36.8	NS	NS	3	FSL
Mandelli et al. (2016)	bvFTD vs. Con	34	62.3 ± 6.6	23	62.9 ± 6.5	NS	NS	26.6 ± 3.5	1.5/3	SPM8
Melloni et al. (2016)	bvFTD vs. Con	22	68.3 ± 5.8	26	68.0 ± 11.4	NS	NS	NS	1.5	SPM12
Buhour et al. (2017a)	bvFTD vs. Con	15	66.5 ± 8.3	15	67 ± 8.2	NS	NS	NS	1.5	SPM5
Bertoux et al. (2018)	bvFTD vs. Con	20	68.9^#^	12	68.3^#^	NS	NS	NS	1.5	FSL

*bvFTD, behavioral variant frontotemporal dementia; ALS, amyotrophic lateral sclerosis; ALSFRS-R, revised ALS functional rating scale; NS, not specified; Superscript: a, ALS with right dominant; b, ALS with left dominant; c, clinical dementia rating (CDR) = 0.5; d, CDR = 1; e, CDR = 2–3*;

* *, the range was provided*;

# *, SD was not provided*.

### Single-Dataset Analysis

**Figure 2:**
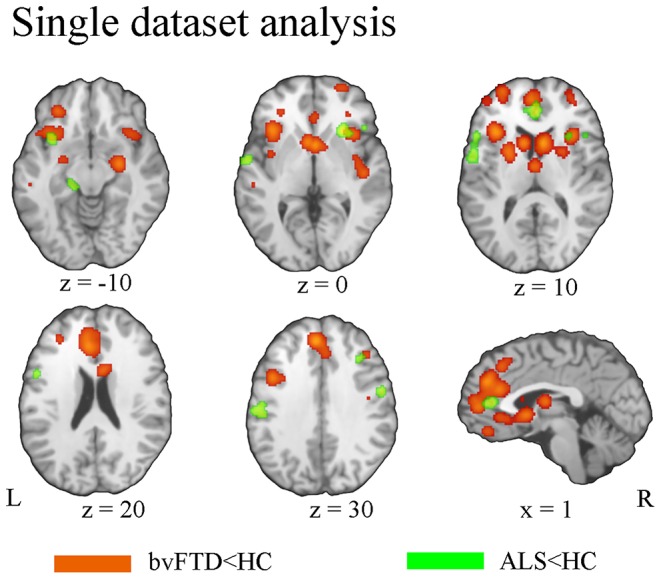
Pattern of gray matter atrophy in bvFTD (orange) and ALS (green) based on single-dataset analysis.

BvFTD patients demonstrated gray matter atrophy in extensive brain areas involving the anterior cingulate cortex (ACC), medial frontal gyrus, left inferior orbitofrontal gyrus, gyrus rectus, and bilateral anterior insula extending to the inferior frontal gyrus. Besides, gray matter atrophy was also identified in subcortical regions, involving the bilateral caudate, putamen, and thalamus (Figure 2). Patients with ALS exhibited gray matter atrophy in the bilateral motor cortex, inferior frontal junction area, superior temporal gyrus, and ACC (Figure 2).

#### Conjunction and Contrast Analysis

**Figure 3:**
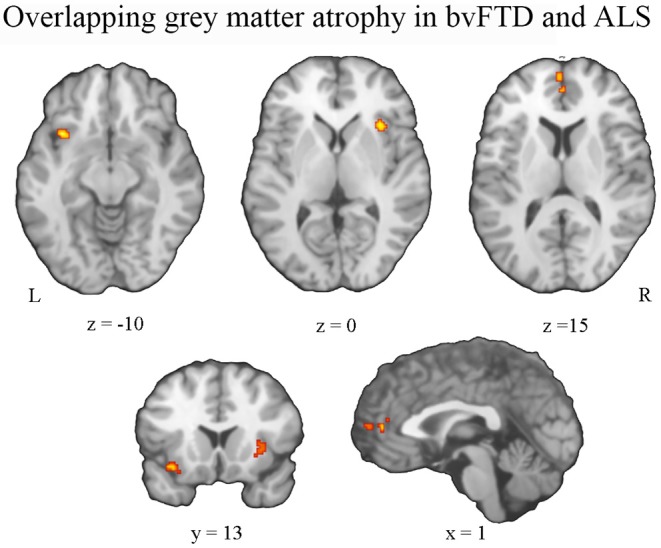
Pattern of overlapping gray matter atrophy between bvFTD and ALS. An overlap of gray matter atrophy in frontolimbic regions involving the bilateral anterior insula and anterior cingulate cortex was revealed.

**Figure 4:**
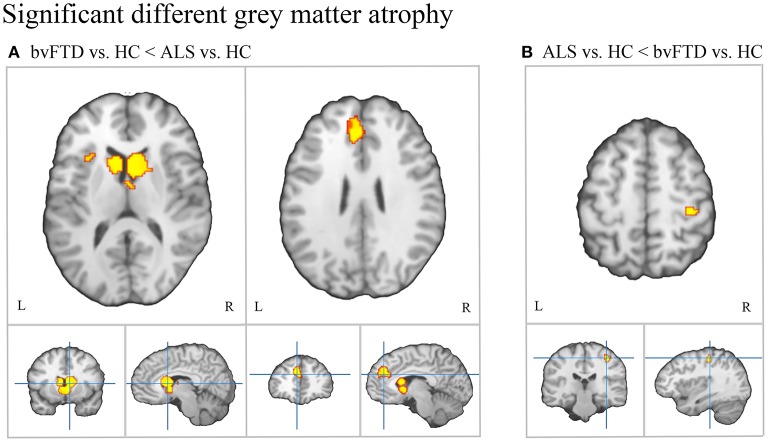
Patterns of distinct gray matter atrophy between bvFTD and ALS. **(A)** Significantly greater gray matter atrophy in the medial frontal/anterior cingulate cortex, bilateral caudate, and left anterior insula in bvFTD relative to ALS. **(B)** Significantly greater gray matter atrophy in the right motor cortex in ALS relative to bvFTD.

Conjunction analysis revealed convergent atrophy between bvFTD and ALS in frontolimbic structures involving the bilateral anterior insula and ACC (Figure 3, Table 2A). The subtraction of the first-level bvFTD and ALS ALE results revealed greater gray matter atrophy in the medial frontal cortex/ACC, bilateral caudate, left anterior insula, and right thalamus in bvFTD relative to ALS (Figure 4A, Table 2B) and greater gray matter atrophy in the right motor cortex in ALS relative to bvFTD (Figure 4B, Table 2C).

**Table 2:** Clusters demonstrating overlapping and distinct patterns of gray matter atrophy in ALS and bvFTD.

**Cluster**	**Volume (mm^**3**^)**	**Hemisphere**	**Label**	**Talairach coordinates of peak voxel**			**BA**
				**X**	**Y**	**Z**	
**A. Overlapping atrophy in bvFTD and ALS**							
1	464	R	Insula	32	16	2	13
2	168	L	Insula	−34	12	−10	13
3	80	L	Medial frontal gyrus	0	54	10	10
4	56	R	Anterior cingulate	2	44	8	32
**B. Greater atrophy in bvFTD**							
1	5,264	L	Anterior cingulate	3.9	6.9	4.2	25
		L	Caudate	7.2	11	13.8	/
2	1,664	L	Anterior cingulate	−3.6	37.9	27.6	32
3	1,208	L	Caudate	−7.5	11.8	12	/
4	576	L	Insula	−31	14.6	6.4	13
5	128	R	Thalamus	3.3	−5.3	12	/
**C. Greater atrophy in ALS**							
1	360	R	Postcentral gyrus	36.9	−28.2	48.5	2

#### Analysis of Behavioral Domain Profiles

**Figure 5:**
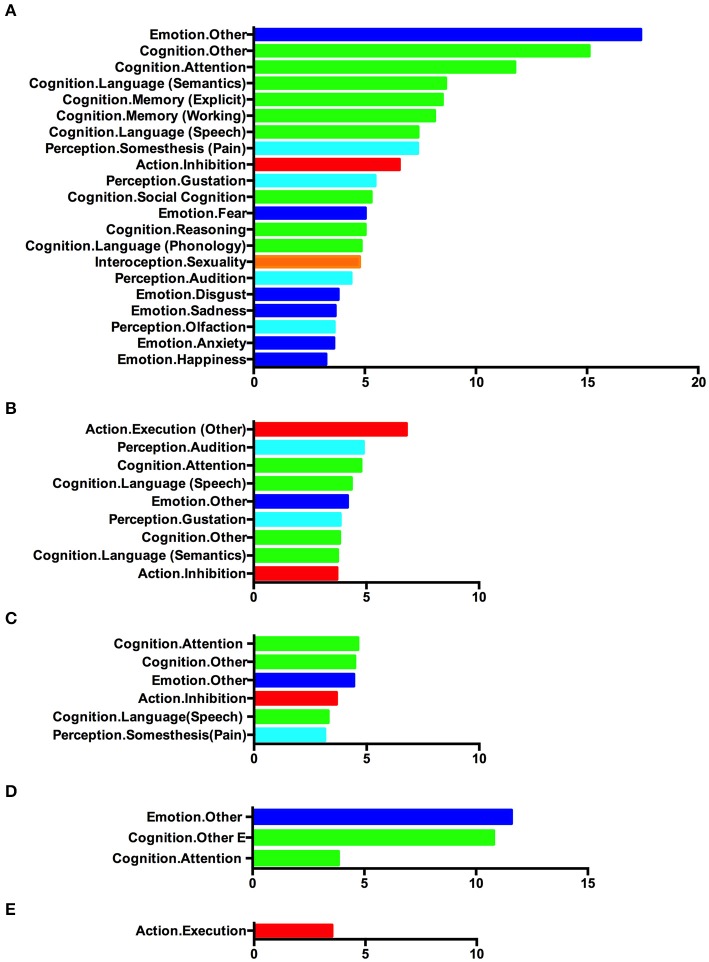
Behavioral characterization of the significant atrophic regions identified in **(A)** single-dataset analysis based on bvFTD vs. control foci, **(B)** single-dataset analysis based on ALS vs. control foci, **(C)** conjunction analysis showing overlap between bvFTD and ALS, **(D)** contrast analysis showing greater atrophy in bvFTD, and **(E)** contrast analysis showing greater atrophy in ALS. Only behavioral domains significantly associated with the respective clusters at *p* < 0.05 (corrected for multiple comparisons) are illustrated.

To assess the functional roles of these atrophic regions, we performed behavioral analysis using the BrainMap database. The results are illustrated in Figure 5. As illustrated, the regions affected in bvFTD patients were mainly associated with various emotional and cognitive processes. Besides, the perception/interception and inhibition functions also showed a significant association with those regions (Figure 5A). The atrophic regions affected by ALS showed a significant association with motor execution, attention, language-related processing, and emotion (Figure 5B).

Analysis of the behavioral correlates was also conducted on the atrophic regions identified in the conjunction/contrast analysis. Clusters with convergent gray matter atrophy between bvFTD and ALS patients were mainly associated with cognitive function, emotion, inhibition, and somatosensory processing (Figure 5C). Regions showing greater atrophy in bvFTD demonstrated a strong connection with emotional and cognitive processes (Figure 5D). Clusters with significantly greater atrophy in ALS showed a significantly high association with motor execution (Figure 5E).

## Discussion

In the present study, we performed a meta-analysis of VBM studies that evaluated the pattern of gray matter atrophy in ALS or bvFTD by comparing each patient population to their controls and contrasted the patterns of gray matter atrophy in bvFTD and ALS patients to identify the commonality and differences in neural signature. Our results outlined a convergence of gray matter atrophy in the frontolimbic structures involving the bilateral anterior insula and ACC, which were accompanied by specific and distinct atrophy within the frontomedial/ACC-caudate regions in bvFTD patients and right motor cortex in ALS patients. Besides, we also applied a data-driven approach by calculating behavioral domain profiles for atrophic regions detected in the ALE meta-analysis to relate those neural changes to clinical impairments. Rather than discussing results simply by reviewing the literature, this data-driven approach refrains from the bias of previous meta-analysis associated with subjective presumptions and domain-specific problem (Laird et al., 2009; Lancaster et al., 2012).

By comparing the foci identified in the studies that compared bvFTD patients and controls and those that compared ALS patients and controls, our study revealed a convergent spatial pattern of gray matter reduction in frontolimbic structures involving the ACC and anterior insula. A convergent pattern of gray matter atrophy in bvFTD and ALS patients in the ACC has been reported previously (Lillo et al., 2012a; Crespi et al., 2018). Additionally, previous meta-analyses that reviewed the separate literature of VBM studies of bvFTD (Pan et al., 2012; Schroeter et al., 2014) and ALS (Shen et al., 2016) have reported insula involvement in both diseases. Though ALS is referred to as a motor disease, deficits in fluency, executive function and social cognition have been shown (Elamin et al., 2012; van der Hulst et al., 2015; Beeldman et al., 2016, 2018), sharing similarities with behavioral changes and cognitive decline in bvFTD. Our results are consistent with those previous findings and support the notion of convergent pathological processes between ALS and bvFTD by pulling together all the published results from different VBM studies of bvFTD and ALS.

In addition to the detection of spatial convergence of neuro-structural degeneration, our study also revealed greater atrophy in the frontomedial-caudate circuit in bvFTD relative to ALS. Atrophy in the medial frontal regions has been consistently reported by meta-analyses on gray matter atrophy pattern in bvFTD (Pan et al., 2012; Schroeter et al., 2014). Although cortical atrophy has been described extensively in the frontal and polar temporal regions, the atrophy of the medial prefrontal cortex has been shown to be specific to bvFTD (Salmon et al., 2003) and occurs even in the early course of illness (Seeley et al., 2008). In addition to the cortical regions, the subcortical regions, including the caudate and thalamus, are also affected early in bvFTD (Garibotto et al., 2011; O'Callaghan et al., 2014; Macfarlane et al., 2015). There is increasing evidence from functional neuroimaging studies of the healthy that striatal regions modulate behavior and cognition (O'Callaghan et al., 2014), impairments of which are the manifestations of bvFTD. Together with those findings, our results highlighted the frontomedial/ACC-caudate circuit as the core anatomical correlate of bvFTD, which discriminates it from typical ALS patients. The combined atrophy of frontomedial/ACC and striatal regions might provide better neuroimaging biomarkers for bvFTD pathology.

Interestingly, the core areas of bvFTD that showing prominent atrophy are located close to regions with convergent gray matter atrophy between bvFTD and ALS. It has been revealed that ALS patients who scored significantly lower in social cognitive performances during follow-up, exhibited abnormal functional connectivity at baseline within cognitive networks similar to those described in early bvFTD, including frontolimbic networks (Trojsi et al., 2015, 2017). We speculate that early damage of these overlapping regions makes ALS patients more prone to developing cognitive and/or behavioral impairments in the later stages of the disease, and FTD development in ALS is likely to be accompanied by progressive atrophy in the frontomedial-caudate circuit. Accordingly, Masuda and colleagues found that patients with ALS-FTD showed the greatest atrophic changes in the caudate head and medial frontal gyrus (Masuda et al., 2016), indicating that the caudate head and its networks were most vulnerable to lesion in sporadic ALS-FTD-spectrum patients and are associated with cognitive decline with FTD features. Future longitudinal studies are needed to examine whether this gray matter atrophy pattern and its degree of severity were related to an increased probability of FTD development in ALS patients.

BvFTD is clinically characterized by progressive changes in personality, social behavior, and cognition (Piguet et al., 2011), with specific features including apathy, loss of empathy, disinhibition, hyperorality, perseverative behavior, and executive deficits (Rascovsky et al., 2011). Indeed, analysis of behavioral domain profiles confirmed that the affected networks in bvFTD were related to various kinds of emotional and cognitive processing, as well as inhibition and pain perception, mirrored the clinical deficits in bvFTD. In particular, those atrophic regions were highly associated with emotional processing. Intact emotional abilities are a premise for empathy, the ability to infer another's emotional state. It is well-acknowledged that loss of empathy is an early and central symptom of bvFTD (Rascovsky et al., 2011). Besides, emotion recognition (e.g., emotion recognition through facial and vocal stimuli) has also been posited to contribute to social cognition (Petroni et al., 2011), which has been conceptualized as fundamental deficits of changes in interpersonal behavior and personality. Impairments in emotional processing could be one of the core pathological changes underlying clinical deficits observed in bvFTD.

Unlike the extensive pattern of gray matter atrophy in bvFTD patients, ALS patients presented with a relatively limited pattern of gray matter atrophy. This could be attributed to the fact that most of the original VBM studies included in the present study enrolled ALS patients that were at a relatively mild stage of the disease without evident dementia. Actually, in the present meta-analysis, the disease duration of ALS patients in the included studies was generally shorter than that of bvFTD patients. In spite of the inherent heterogeneity of the ALS patients, structural and functional abnormalities of the motor cortex in ALS patients have been consistently identified by various imaging modalities (Agosta et al., 2012; Cosottini et al., 2012; Foerster et al., 2012; Verstraete et al., 2012) and also validated by meta-analyses (Sheng et al., 2015; Shen et al., 2016). Regional gray matter loss in the motor cortex is likely to reflect the degeneration of giant pyramidal cells in the motor cortex, which has been regarded as the pathological hallmark of ALS patients. Not surprisingly, our study found greater atrophy in the right motor cortex in ALS patients relative to bvFTD patients. This region plays a key role in the motor system, consistent with the fact that motor execution is the most prominent clinical symptoms of ALS. Our results confirmed gray matter atrophy in motor regions to be the most prominent and replicable gray matter abnormality in ALS. Gray matter atrophy in the motor cortex could be a potential reliable marker for upper motor neuron involvement in ALS patients and may supplement clinical examination.

According to neuropathological studies, TDP-43 pathology in ALS disseminates from the motor cortex to prefrontal areas and finally reaches the anteromedial portions of the temporal lobe (Brettschneider et al., 2013). In contrast, neuropathological studies of bvFTD suggested that disease originates in the anterior prefrontal cortex, extends to the middle frontal and temporal regions, and later involves the sensorimotor cortex followed by the visual cortex (Brettschneider et al., 2014). The atrophy patterns identified in ALS and bvFTD by the present meta-analysis are consistent with the pattern of pathological changes. It has been proposed that pathological propagation of “prion-like” protein is at the root of some neurodegenerative diseases (Soto, 2012) and that transmission of TDP-43 pathology along axonal pathways represents the underlying mechanism of the stereotyped progression of the ALS-FTD spectrum (Agosta et al., 2015, 2016). Our results concerning convergent and distinct patterns of gray matter atrophy in bvFTD and ALS could represent the different phenotypical expressions of the same neurodegenerative process that is associated with the spreading pattern of TDP-43 pathology.

There are several limitations. Firstly, the voxel-wise meta-analysis was based on the pooling of peak stereotactic coordinates from the original studies rather than on raw statistical brain maps, which could lead to less accurate results. Secondly, the ALE method does not take non-significant findings into account and may give the wrong impression of consistency across studies. Attention should be paid when interpreting the results in ALS patients, as significant brain atrophy was not consistently found. Thirdly, the heterogeneity of the methodologies (i.e., different reprocessing protocols and statistical thresholding methods) in the VBM studies may yield an inappropriate combination of results across studies. Lastly, factors such as age, genetics, and clinical subtype can affect the topography of gray matter degeneration. Notably, in the current study, subjects in the bvFTD literature were generally older than subjects in the ALS literature. Because the aim of this study was to identify the commonality and differences in the pattern of gray matter atrophy between bvFTD and ALS by comparing the two sets of differences in gray matter volume between patients and their age-matched controls, the potential effect of unmatched age between the bvFTD literature and ALS literature on our results is limited. Regardless, it would be interesting to know how these factors modulate the degeneration topography, and future experimental work is warranted to probe these associations.

Despite those limitations, our findings—drawing upon a large number of reports from both bvFTD and ALS studies—provided important new information on how these disease states diverge and how they may converge upon each other. First, we demonstrated a convergent gray matter atrophy pattern between bvFTD and ALS involving the frontolimbic regions and supporting the presence of subtle bvFTD-like deficits in ALS patients. Besides, we also identified a disease-specific pattern of gray matter degeneration, with a greater degree of atrophy in the frontomedial-caudate circuit in bvFTD patients and a higher degree of atrophy in the motor cortex in ALS patients, which underpinned the clinical manifestations of those two diseases.

## Data Availability Statement

All datasets generated for this study are included in the article/supplementary material.

## Author Contributions

SL planned the study. CL and NH reviewed the literature. CL, NH, YX, and WZ analyzed the data. CL wrote the article. QG and SL edited the paper. All the authors contributed to manuscript revision and approved the final version of the article.

### Conflict of Interest

The authors declare that the research was conducted in the absence of any commercial or financial relationships that could be construed as a potential conflict of interest.
